# Life-threatening hypotension in the immediate postoperative period of cataract surgery under topical anesthesia: a report of two cases

**DOI:** 10.1186/s12871-022-01894-0

**Published:** 2022-11-11

**Authors:** Michele Fostier, Gintare Januleviciute, Fabrice Fauconnier, Edith Collard, Virginie Dubois

**Affiliations:** Anesthesiology Department CHU UCL Namur Site Godinne, Av. G. Therasse 1, 5530 Yvoir, Belgium

**Keywords:** Cataract surgery, Systemic adverse events, Anesthesia care, Topical anesthesia, Ambulatory surgery

## Abstract

**Background:**

Cataract surgery is one of the most frequent surgeries in the world. It is a very safe procedure mostly performed under topical anesthesia in outpatients centers. Due to the growing lack of anesthesiologists, cataract surgeries are more frequently performed without an anesthesiologist present in the operating room. Although extremely rare, life-threatening complications may occur.

**Cases presentation:**

We report two cases of cataract surgery complicated by severe hypotension that required emergency resuscitation in the immediate postoperative period and hospitalization in intensive care unit. Anaphylactic shock was confirmed in the first case and suspected in the second.

**Conclusions and importance:**

Even though cataract surgery is a very safe procedure, it is essential to ensure the presence of an anesthesiologist to manage potential, though extremely rare, life-threatening complications such as anaphylactic reactions.

## Background

Cataracts are the leading cause of blindness worldwide, affecting 20% of people aged 65 years and 60% of those older than 85 years. Globally, an estimated 94 million people are visually impaired and 20 million are blind due to cataracts [1]. The only effective treatment to restore vision is cataract surgery [2], which is the most performed surgical procedure globally. In the last few decades, technological advances in phacoemulsification, foldable intraocular lenses, and surgical safety procedures have allowed cataract surgery to be performed essentially in ambulatory centers. Topical anesthesia with eye drops is now the most widely used method; hence, the need for an anesthesiologist during the procedure is rare.

Cataract surgery is a very safe procedure with few patients experiencing serious sight-threatening adverse events [3] and has an estimated morbi-mortality rate between 0.01% and 0.05% [2], which is similar to the risk for the general population. Therefore, more than 99% of cataract surgeries are performed in an outpatient setting [4] and more frequently without the presence of an anesthesiologist. Office-based cataract surgeries with no anesthesiologist available have also been reported [4].

Even though the rate of systemic complications after cataract surgery is extremely low, the following two cases highlight the importance of having an anesthesiologist present to managing potentially life-threatening complications.

## Case presentation

In our institution, all cataract surgeries are performed in the ambulatory center. Patients are asked to complete a health questionnaire, and most of them are seen for a preoperative evaluation. Patients may or may not be asked to fast prior to surgery, depending on the surgeon. Usual medications, including anticoagulants, are continued. An intravenous line is placed on arrival and an anesthesiologist (assistant or nurse anesthesiologist always supervised by a senior) is present in the operating room.

### Case 1

The first patient was an 81-year-old woman classified per the American Society of Anesthesiologists (ASA) Physical Status Classification System as ASA II due to hypothyroidism, hypertension, pulmonary embolism, gastric ulcer, and hepatitis B. She had no history of allergy. She underwent an uncomplicated cataract surgery for the first eye on September 7, 2020, and she presented 2 weeks later for the second eye. According to her surgeon, she did not fast. The surgery was performed uneventfully and no intervention by the anesthesiologist was necessary. Postoperatively, while escorting the patient toward her room, she complained of chest pain and discomfort before falling unconscious with urinary incontinence. Blood pressure (BP) and heart rate (HR) were measured at 40/20 mmHg and 140 beats per minute, respectively. She was immediately managed by the anesthetic team by orotracheal intubation, intravascular filling and norepinephrine support. She was then admitted to the intensive care unit (ICU) where she was noted to have palpebral and labial edema and lower limbs rashes. After 24 h, the vasopressor support was weaned, and the patient was extubated; she was discharged the following day with a 7-day course of antibiotics for a suspected aspiration pneumonia as she did no fast. Serum Tryptase, a specific marker for anaphylaxis, was significantly increased. An anaphylactic shock due to a substance received during the surgery was suspected. Therefore, three months later she underwent cutaneous allergic tests in day hospital close medical supervision and all medications used during surgery were tested. She rapidly developed another anaphylactic shock, which was treated effectively by epinephrine and corticosteroids. An allergy to cefuroxime was confirmed. This antibiotic is administered intraocularly at the end of each cataract surgery to prevent endophthalmitis, and we believe that sensitization to this molecule occurred during the first surgery leading to anaphylactic shock at the second operation.

### Case 2

The second patient was a 77-year-old healthy woman classified as ASA II due to hypothyroidism and rhizomelic pseudo-polyarthritis treated with 4 mg methylprednisolone (Medrol). She had no history of allergy. Cataract surgery for the first eye was performed three weeks before, without any complications, and she presented for the second eye. According to her surgeon, she fasted. The surgery completed without complication and she was escorted to her room where she received a light meal before leaving. Thirty minutes after surgery she felt discomfort with nausea. BP was 60/30 mmHg and HR was 30 per minute. Atropine (0.25 mg) was administered through the intravenous line and her BP and HR increased to 90/50 mmHg and 80 per minute, respectively. She was admitted in the Postoperative Acute Care Unit where she developed vomiting, agitation, and a sudden desaturation to 88% under 6 L of oxygen while BP and HR normalized. Her chest radiograph showed a small right perihilar infiltrate. A chest CT with contrast was performed, which revealed bibasal pneumonitis. COVID-19 investigations were negative. Cardiac echography was normal. After a few hours of noninvasive ventilation, she recovered and oxygen saturation normalized. She fully recovered and was discharged 3 days later. Serum Tryptase was not significantly high but unfortunately, the patient did not wish to perform any other tests to confirm the anaphylactic reaction hypothesis.

## Discussion and conclusions

Cataract surgery is the most performed surgery worldwide. Due to recent technological advances and improvements in the safety of the procedure, most surgeries are now performed using topical anesthesia with eye drops; therefore, anesthesiologists are rarely required during the procedure. Murray et al. [5] reported only one serious adverse event in a series of 6661 cataract surgeries and concluded that most cataract surgery procedures under local anesthesia could be safely performed without the presence of an anesthesiologist.

Between January 1, 2017 and December 31,2021 we performed 5943 cataract surgeries in our institution including 5369 cases under topical anesthesia and 574 cases under general anesthesia. We did not encounter any serious complication apart the cases described here in two healthy patients. We presented two cases of life-threatening adverse events associated with cataract surgery; resuscitation was needed in the first patient and ventilation support for the second with a favorable outcome for both patients thanks to the immediate intervention of the anesthetist team. An anaphylactic reaction was confirmed in the first case, but this result was less clear in the second case, even though the clinical manifestations suggested an anaphylactic reaction, the patient did not wish to undergo allergy tests.

Anaphylactic reaction following intracameral cefuroxime injection is extremely rare. To the best of our knowledge, only one case has been reported in the literature of a patient with a history of penicillin allergy [6]; this was not the case in our first patient. In accordance with Moissaiev and Levinger [7], we believe that ophthalmologists performing cataract surgery should be aware of this very rare life-threatening complication and that an anesthesiologist should be present in or near the operating room. Systemic complications such as hypertension and agitation are frequent and may require the intervention of the anesthesiologist [8].

Our two patients also presented with pneumonitis probably caused by inhalation. Hence, we recommend that patients fast for at least 6 h for solids and 2 h for liquids prior to cataract surgery [9].

Perioperative anesthetic care in cataract surgery is still debated and practices vary worldwide. Even though several studies have been published on the safety of a protocol without an anesthesiologist present in the operating room, we recommend cataract surgery be performed for patients who fasted, in a center with immediate availability of an anesthesiologist because life-threatening complications, such as severe anaphylactic reactions, may require immediate intervention by resuscitation that cannot be managed by ophthalmologists alone. We therefore do not recommend office-based cataract surgeries without an anesthesiologist present.

## Data Availability

The data analyzed during the current study available from the corresponding author on reasonable request.

## Abbreviations

ASA: American Society Of Anesthesiologists
HR: Heart Rate
BP: Blood Pressure
ICU: Intensive Care Unit
CT: Computed Tomograpgy
