# Correction: Life-threatening hypotension in the immediate postoperative period of cataract surgery under topical anesthesia: a report of two cases

**DOI:** 10.1186/s12871-023-02087-z

**Published:** 2023-04-14

**Authors:** Michele Fostier, Gintare Januleviciute, Fabrice Fauconnier, Edith Collard, Virginie Dubois

**Affiliations:** Anesthesiology Department, CHU UCL Namur Site Godinne, Av. G. Therasse 1, Yvoir, 5530 Belgium

**Correction: BMC Anesthesiology 22, 345 (2022)**

**https://doi.org/10.1186/s12871-022-01894-0**

Following the publication of the article [1], it appeared that a bibliographical reference about the first case described [2] had been omitted. We apologize for any inconvenience caused by this error.

## References

[1] Fostier M, Januleviciute G et al. Life-threatening hypotension in the immediate postoperative period of cataract surgery under topical anesthesia:a report of two cases. BMC Anesthesiology. 2022;22:345. 10.1186/s12871-022-01894-0 10.1186/s12871-022-01894-0 PMC9650168 36368969

[2] Mahiat C, Robaye S (2022). Anaphylactic shock following cataract surgery: a documented intracameral cefuroxime allergy. J Investig Allergol Clin Immunol.

